# Bexarotene: A Rare Cause of Misleading Thyroid Function Tests

**DOI:** 10.7759/cureus.11591

**Published:** 2020-11-20

**Authors:** Vishwanath Pattan, Kira Schaab, Vishnu Sundaresh

**Affiliations:** 1 Endocrinology, Wyoming Medical Center, Casper, USA; 2 Family Medicine, The University of Wyoming Family Medicine Residency Program-Casper, Casper, USA; 3 Endocrinology, University of Utah Health, Salt Lake City, USA

**Keywords:** central hypothyroidism, bexarotene, mycosis fungoides, cutaneous t-cell lymphoma

## Abstract

Bexarotene is a very rare cause of central hypothyroidism (CH) and its effects have been reported to be dose-dependent; however, the available data in the literature on dose-dependent effects are variable. The standard practice of monitoring thyroid function using thyroid-stimulating hormone (TSH) to adjust levothyroxine (LT4) dose does not apply to bexarotene since it causes CH. In CH, TSH is not reliable. Hence free tetraiodothyronine (fT_4_) level is used to monitor and adjust the LT4 dose. We report a case of an 81-year-old Caucasian male with cutaneous T-cell lymphoma (CTCL) who was treated with bexarotene. His pre-treatment TSH was normal at 1.6 µIU/mL (reference range: 0.46-4.68 µIU/mL). Post-bexarotene, the total tetraiodothyronine (T_4_) level was within the reference range, but a downward trend was noted. Eventually, total triiodothyronine (T_3_) dropped to a low level of 0.61 ng/mL (reference range: 0.97-1.69 ng/mL), and LT4 was initiated. Bexarotene dose was increased, but LT4 was not increased by the primary physician who relied on TSH level, which was low, and hence the existing LT4 dose was maintained. The patient had persistent symptoms of hypothyroidism and, eventually, a diagnosis of CH was made. The symptoms of hypothyroidism improved after normalizing fT_4_, with an increase in the LT4 dose. This case represents an example of missed CH due to bexarotene, ­which led to suboptimal LT4 replacement impacting the quality of life for the patient.

## Introduction

The standard practice in the management of primary hypothyroidism involves monitoring of thyroid-stimulating hormone (TSH) to guide dose adjustments for levothyroxine (LT4). However, an exception to this is central hypothyroidism (CH), which is caused mostly by pituitary tumor/surgery and drugs. In this situation, TSH is unreliable and hence free tetraiodothyronine (fT_4_) is used for monitoring and adjusting the LT4 dose. The goal set for fT_4_ is just above the 50th percentile of the normal reference range (0.8-2.19 ng/dL) to achieve a good quality of life. Recognizing the drugs that can cause CH and changing the practice of hypothyroidism management accordingly are important to prevent therapeutic mishaps. Bexarotene is a synthetic retinoid, which selectively activates the retinoid X receptor [1]. Bexarotene is a rare but well-recognized cause of CH, and it has been reported to cause dose-dependent suppression of TSH [2]. In the last decade, several new drugs have been reported to cause CH, and providing continuing medical education to primary care physicians is very important. In this case report, we discuss the challenges of diagnosing and managing bexarotene-induced CH in the primary care setting.

## Case presentation

**Figure 1:**
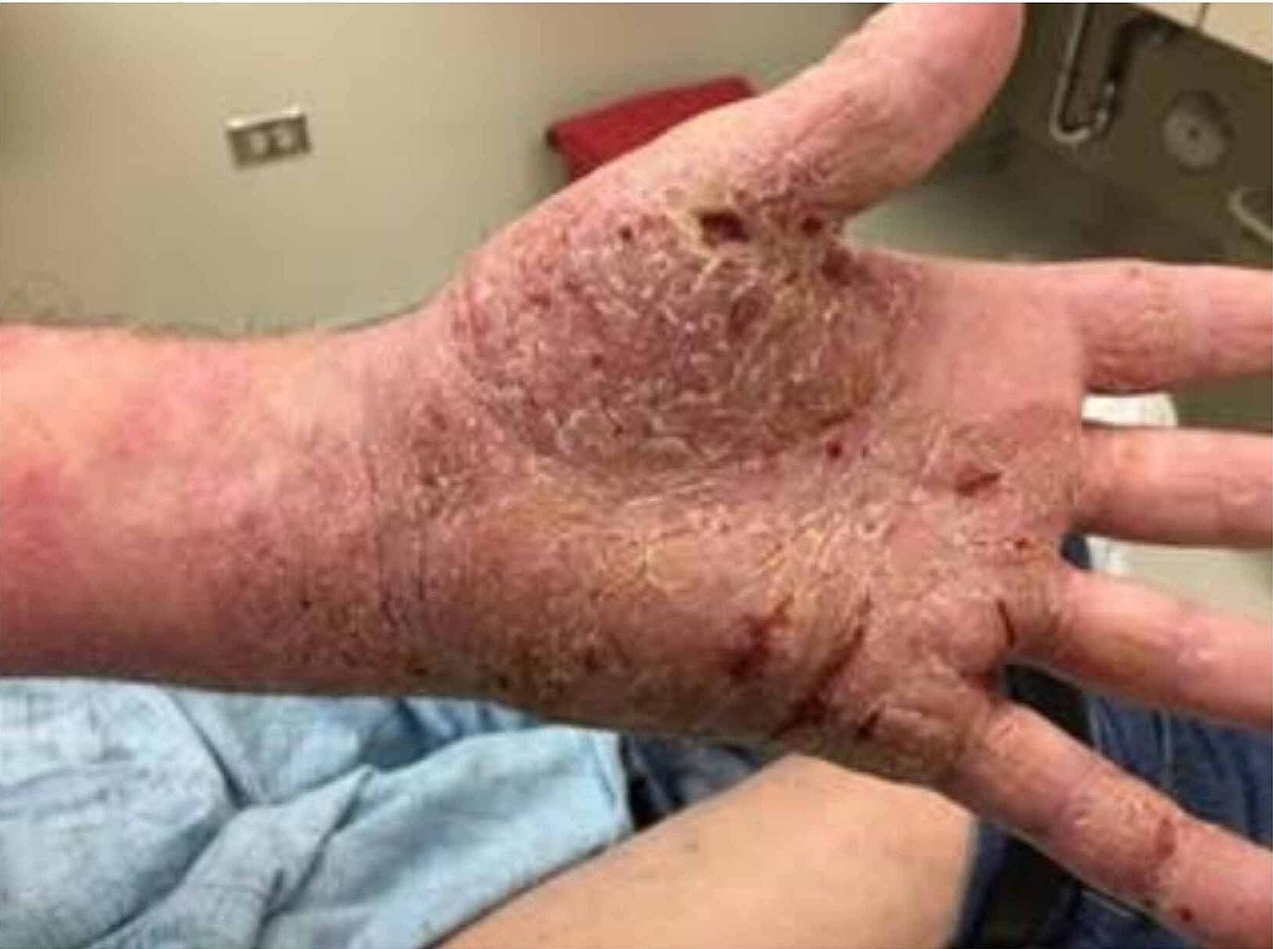
Initial rash of mycoses fungoides on the left palm and wrist

An 81-year-old Caucasian male presented to the endocrinology office for the management of hypothyroidism in February 2020. The patient had initially presented in 2014 with a rash on the left palm and wrist (Figure 1), and right thigh. He had undergone a biopsy and had been diagnosed with cutaneous T-cell lymphoma (CTCL) (mycosis fungoides). He had been started on bexarotene 150 mg (two tablets of 75 mg each) daily in August 2015, and the dose had been temporarily increased to 300 mg (two tablets of 75 mg each) daily during disease exacerbation. Bexarotene had been tapered down to 150 mg per day whenever the flares had resolved. His baseline TSH had been 1.6 µIU/mL (reference range: 0.46-4.68 µIU/mL). He had follow-up labs ordered for total tetraiodothyronine or total T_4_ by the oncologist, which had been normal until March 2016 but showed a downward trend. In April 2016, total triiodothyronine or total T_3_ had been low at 0.61 ng/mL (reference range: 0.97-1.69 ng/mL), and LT4 50 µg daily had been initiated by the oncologist (Table 1). Subsequently, the patient had been followed up by the primary care physician for the management of hypothyroidism.

Lab reviews had revealed that the T_4_ or fT_4_ decreased when the dose of bexarotene was increased (all available labs are summarized in Table 1). Conversely, the T_4_ or fT_4_ levels increased when bexarotene was decreased or stopped. The patient had been maintained on LT4 50 µg daily until March 2017 when both bexarotene and LT4 had been held during the hospitalization for sepsis due to pneumonia. This discontinuation had normalized the fT_4_ levels to 1.13 ng/dL in March, and to 1.3 ng/dL in April 2017. Although bexarotene had been restarted after three weeks in April 2017, LT4 had not been restarted until May 2017. By May 2017, fT_4_ had dropped to 0.5 ng/dL and LT4 75 mcg was restarted. The patient had persistently low T_4_, fT_4_, and T_3_ values, but LT4 had not been increased beyond 75 µg daily, because of concerns related to suppressed TSH.

**Table 1:** Serial thyroid lab panel and the corresponding doses of levothyroxine and bexarotene ^¥^Higher dose during the flare (N): lab value is within the normal range; (L): lab value is below the normal range; TSH: thyroid-stimulating hormone; T_3_: triiodothyronine; T_4_: tetraiodothyronine

Date	Free T_3_ (reference range: 2.77-5.27 pg/mL)	Total T_3_ (reference range: 0.97-1.69 ng/mL)	Free T_4_ (reference range: 0.8-2.19 ng/dL)	Total T_4_ (reference range: 5-12 µg/dL)	TSH (reference range: 0.46-4.68 µIU/mL)	Daily bexarotene dose (mg)	Levothyroxine dose (µg)
6/3/2014					1.60 (N)		
2/3/2016				6.4 (N)		150 to 300^¥^	
3/17/2016				5.3 (N)		300	
4/15/2016		0.61 (L)		5.6 (N)		150 to 300^¥^	50
7/11/2016		0.89 (L)		7.9 (N)		300	50
8/1/2016	3.26 (N)			7.7 (N)		300	50
9/9/2016	2.66 (L)		1.14 (N)			300	50
10/5/2016		0.57 (L)		6.0 (N)		300	50
11/7/2016		0.874 (L)	1.15 (N)	7.7 (N)		300	50
1/23/2017		1.01 (N)		8.2 (N)		300	50
3/7/2017			1.13 (N)			Stopped for 3 weeks	Stopped
4/3/2017			1.30 (N)		<0.015 (L)	300	Stopped
4/6/2017			0.82 (L)		<0.015 (L)	300	Stopped
5/4/2017			0.50 (L)		0.127 (L)	300	75 (restarted)
5/23/2017			0.52 (L)			300	75
6/21/2017	1.94 (L)		0.56 (L)			Decreased to 225 mg	75
7/31/2017	2.62 (L)		0.61 (L)			225 mg	75
10/9/2017	2.49 (L)		0.85 (L)		<0.015 (L)	300 (increased to 375 mg in December for flare)	75
1/2/2018	2.45 (L)		0.76 (L)			300	75
3/7/2018	2.67 (L)		0.83 (L)			300	75
4/30/2018	1.81 (L)		0.72 (L)			300	75
7/20/2018	2.89 (N)		0.81 (L)			375	75
9/14/2018	3.20 (N)		0.62 (L)			300	75
4/12/2019	2.21 (L)		0.77 (L)			300	75
8/20/2019	2.44 (L)		0.70 (L)			375	75
1/27/2020	2.25 (L)		0.59 (L)			450	Increased from 75 to 100 (on 2/18/2020)
3/3/2020	2.01 (L)		0.60 (L)			450	Increased from 100 to 112
7/20/2020	1.36 (L)		0.7 (L)		0.089 (L)	450	Increased from 112 to 150
8/10/2020	2.37 (L)		1.1 (N)		<0.015 (L)	450	150

The patient was initially seen by the endocrinologist in February 2020 because of a persistent abnormal thyroid function test. He was not using biotin and had not received iodinated contrast previously. Pertinent review of symptoms included decreased exercise tolerance, muscle aches and pain in the extremities, decreased appetite, cold intolerance, and easy bruising. On physical examination, the thyroid was normal in size, with no palpable nodules and no bruit on auscultation.

Additional labs were ordered for screening of pituitary hormonal function, which were normal: alpha subunit: 0.3 ng/mL (reference range: 0-0.4 ng/mL), prolactin: 12.5 pg/mL (reference range: 4-15.2 pg/mL), adrenocorticotropic hormone (ACTH): 22 pg/mL (reference range: 7.2-63 pg/mL), and cortisol: 14.8 µg/dL (reference range: 4.46-22.7 µg/dL). MRI brain/pituitary showed a normal sized pituitary gland without tumor or metastasis.

LT4 was increased to 100 µg daily in February 2020, and a repeat thyroid lab panel two weeks later showed fT_4_ of 0.6 and free T_3_ (fT_3_) of 1.36 (reference range: 2.27-5.27 pg/mL). The LT4 dose was increased to 112 µg daily and a thyroid lab panel was ordered every four weeks. Unfortunately, the patient missed his follow-up labs due to fear and restrictions due to coronavirus disease 2019 (COVID-19) pandemic. LT4 dose was eventually increased to 150 µg daily and then the fT_4_ was found normalized in the follow-up lab done three weeks later. The thyroid lab panel showed fT_4_ of 1.1 ng/dL and fT_3_ of 2.37 with undetectable TSH. The patient endorsed significant improvement in his energy levels with this change, and LT4 150 µg daily was continued. The goal was to slowly titrate the dose up further to bring fT_4_ to the upper half of the reference range. Unfortunately, the patient passed away six weeks later due to acute myocardial infarction.

## Discussion

Bexarotene is a synthetic retinoid, which selectively activates the retinoid X receptor and is used to treat CTCL. It has a peak plasma concentration two hours after ingestion and a half-life of seven hours [1]. Bexarotene selectively inhibits TSH secretion and can therefore lead to CH [2]. In vitro studies have shown that ligands for the retinoid X receptor suppressed the activity of thyrotropin β-subunit gene promoter [3,4]. The decrease in TSH concentrations was reported to be greater in patients who had received higher doses of bexarotene [2]. In phase II and phase III clinical trials in the United States, Duvic et al. (2001) reported that 30‐40% of patients exhibited hypothyroidism at a bexarotene dose of 300 mg/m^2^/day, and approximately 50% of patients exhibited this outcome at doses of more than 300 mg/m^2^/day (i.e., 500 or 650 mg/m^2^/day) [5,6].

According to the United Kingdom consensus statement on safe clinical prescribing of bexarotene, TSH suppression of bexarotene is dose-dependent (Scarisbrick et al., 2013) [7]. Thus, preventive supplementation with LT4 may be appropriate when using bexarotene. It was recommended that LT4 be initiated from day one. LT4 can be started at a low dose of 25-50 µg daily and subsequently titrated to keep the fT_4_ in the upper third of the reference range [7].

The data regarding the dose-dependent drop in TSH and fT_4_ are not consistent. Makita et al. (2019) studied 66 Japanese patients with CTCL on bexarotene [8]. They did not find any dose-dependent effects on TSH and fT_4_ in the dose range of 96-320 mg/m^2^/day. Thus, the dose range of bexarotene and patient ethnicity may influence these effects.

In our patient, the suppression of TSH was observed when increasing the LT4 dose, which was misleading to primary care providers. Earlier detection of the etiology of the patient’s CH may have resulted in more appropriate dose adjustments leading to improved symptoms and better quality of life.

It is important to recognize that changes in bexarotene dose may influence the LT4 dose. LT4 dose requirement may increase with an increase in bexarotene dose. Likewise, the LT4 dose requirement may decrease with a decrease in bexarotene dose or with its discontinuation. Therefore, frequent monitoring of thyroid lab panels and knowledge about changes in bexarotene dose are important. Familiarity and early recognition of bexarotene-induced CH with both TSH and fT_4_ testing can lead to early diagnosis and management of the condition, thereby improving treatment outcomes for the patient.

## Conclusions

Bexarotene causes CH and the effect seems to be dose-dependent. Preventive supplementation with LT4 may be appropriate when using bexarotene. Recognizing the drugs that can cause CH and changing the practice of hypothyroidism management to include a complete thyroid lab panel accordingly are critical to prevent therapeutic mishaps.
